# Neurologists dealing with sickness certification: Experiences of problems and need of competence

**DOI:** 10.1002/brb3.845

**Published:** 2017-10-10

**Authors:** Åsa Snöljung, Jenny Kärrholm, Elin Hinas, Kristina Alexanderson

**Affiliations:** ^1^ Division of Physiotherapy School of Health, Care and Social Welfare Mälardalen University Västerås Sweden; ^2^ Department of Clinical Neuroscience Division of Insurance Medicine Karolinska Institutet Stockholm Sweden

**Keywords:** insurance medicine, neurologist, physician, sick leave, sickness certification

## Abstract

**Rationale and Aims:**

Most studies on physicians' sickness certification practices include general practitioners (GP) while there hardly is any knowledge on this regarding neurologists although neurological diseases often involve work incapacity and need of sick leave.

**Aim:**

The aim was to describe experiences among neurologists in Sweden concerning their work with sickness certification of patients.

**Method:**

A cross‐sectional study of 265 neurologists' responses in a nationwide survey regarding their work with sickness certification of patients was conducted.

**Results:**

The majority (81.5%) had sickness certification consultations at least once a week and a third experienced problems every week in handling sickness certification. Among the 251 who at least sometimes had sickness certification consultations, the following two aspects were experienced as very or fairly problematic: “assess the degree to which the reduced functional capacity limits a patient's capacity to perform his/her work assignments” (67.3%) and “make a long‐term prognosis about the future work capacity of patients on sick leave” (60.5%). At least once a week, 78.7% experienced lack of time regarding managing patient‐related aspects of the sickness certification task. Moreover, 21.8% considered sickness certification to be a work environmental problem, at least once a week. In all, 84% stated that they had a large or fairly large need for more competence concerning sickness certification tasks.

**Conclusions:**

Sickness certification is a common task among neurologists, involving several problematic aspects related to, e.g., lack of competence in assessing function and work capacity and of time. There is a need for improvement.

## 1 INTRODUCTION

Most of the previous studies about sickness certification practices have focused on general practitioners (GP) (Wahlstrom & Alexanderson, 2004; Wynne‐Jones, Mallen, Main, & Dunn, 2010a; Wynne‐Jones, Mallen, Main, & Dunn, 2010b). However, neurologists manage several different patient groups, many of which include patients of working ages, with diseases often leading to work incapacity and need of sickness certification, such as multiple sclerosis, Parkinson's disease, and stroke (Medin, Nordlund, & Ekberg, 2004; Tinghog et al., 2013). With an aging population remaining in paid work, work issues related to neurological diseases increase and knowledge is warranted on the prerequisites for physicians working in neurology clinics for conducting an optimal work with this. Nevertheless, to the best of our knowledge, there are no previous studies focusing on neurologist's work with sickness certification.

All people in Sweden with income from work or unemployment benefits are covered by the sickness absence insurance system. To obtain benefits, the patient must present a medical certificate issued by a physician after the seventh day of a sick‐leave spell. All physicians can issue such certificates, and for many patients with neurological diagnoses, the neurologist remains the treating physician, including handling of sickness certification. Generally, the employer pays benefits during the two first weeks of a sick‐leave spell, thereafter the Social Insurance Agency who also assess if the patient fulfils the criteria for receiving sickness benefits (Wahlstrom & Alexanderson, 2004). Sickness benefits amounts to 80% of lost income, up to a certain level, and can be paid for 1 year and if needed for longer time, even years. After the first 6 months of a sick‐leave spell, the work capacity is to be assessed in relation to work demands of the general labour market.

Consultations where sickness certification is considered involve several different tasks for the physician, including these: assessing whether the patient has a disease or injury; if that diagnosis impairs the patient's functional ability to the extent that the work capacity is also impaired in relation to her or his work demands; together with the patient consider advantages and disadvantages of being on sick leave; prognosticate the duration and degree of work incapacity and needed sick leave; decide on treatments or other measures needed during the sick‐leave period; cooperate with others when needed, e.g., other specialists or stakeholders; issue a sickness certificate; and document actions and plans (Lindholm et al., 2010; Lofgren, Silen, & Alexanderson, 2011; Wahlstrom & Alexanderson, 2004).

When handling these tasks, the physician has to manage not only the role as the patient's treating physician, but also the role as a medical expert giving objective information to other stakeholders. Many physicians find it problematic to handle these two roles (Gulbrandsen, Hofoss, Nylenna, Saltyte‐Benth, & Aasland, 2007; Hussey, Hoddinott, Wilson, Dowell, & Barbour, 2004; von Knorring, Sundberg, Lofgren, & Alexanderson, 2008; Larsson, Sydsjo, Alexanderson, & Sydsjo, 2006; Lofgren, Hagberg, Arrelov, Ponzer, & Alexanderson, 2007; Swartling, Peterson, & Wahlstrom, 2007; Timpka, Hensing, & Alexanderson, 1995; Wahlstrom & Alexanderson, 2004).

According to several studies, one of the most challenging sickness certification tasks for physicians is to assess the degree to which the reduced function actually limits a patient's work capacity (Ljungquist et al., 2015; Nilsson et al., 2012; Ljungquist et al., 2012; Winde et al., 2012; Wynne‐Jones, Mallen, Main, & Dunn, 2010b.

The aims were to describe experiences among specialists and nonspecialists working in neurology clinics concerning their work with sickness certification of patients, regarding frequency of specific situations, perceived problems, need for competence, among all and among.

## 2 METHODS

A cross‐sectional nationwide questionnaire study was conducted including 163 questions about various aspects regarding sickness certification practice. It was sent to 33,144 physicians <68 years of age, working and living in Sweden in 2012 (Ljungquist et al., 2015). The participants were identified by Cegedim AB, a company that manages registries of healthcare staff that also provided information about the physicians' age, gender, and board‐certificated speciality.

The questionnaire was based on two previous surveys, literature reviews, and discussions with clinicians (Lindholm et al., 2010; Ljungquist et al., 2015). Statistics Sweden administrated the questionnaire. In all, 58% responded to the questionnaire and 265 of them (1.5% of all participants) responded that they mainly worked within neurology clinics, hereafter, they are called neurologists. The majority (72.5%) of those neurologists were board‐certified specialists – the rest were under training. In Sweden, to become a specialist requires at least 5 years of resident training, following the initial 5.5 years basic medical education and 2 years of internship.

Answers to the following questions were analysed:

*Frequencies of sickness certification*: “How often in your daily clinical work do you have consultations including consideration of sickness certification?” with response alternatives: More than 10 times a week/6–10 times a week/1–5 times a week/About once a month/A few times a year/Never or almost never (Table 1). Neurologists who did not respond to this question (*n* = 3) or responded “never or almost never” (*n* = 11) were excluded in the following analyses. That is, the 251 neurologists who stated that they had consultations involving consideration of sickness certification at least a few times a year were included in analyses regarding the following items:

**Table 1:** Study population characteristics, gender, age, specialist status, and frequency of sickness certification consultation among physicians mainly working in neurology clinics

	Responding neurologist *n* (%)	Frequency of sickness certification consultations				
		>5 times a week *n* (%)	1–5 times a week *n* (%)	About once a month *n* (%)	A few times a year *n* (%)	Never/ No answer *n* (%)
All	265 (100)	87 (32.8)	129 (48.7)	28 (10.6)	7 (2.6)	14 (5.3)
Gender						
Men	142 (53.6)	54 (38.0)	63 (44.4)	15 (10.6)	2 (1.4)	8 (5.6)
Women	123 (46.4)	33 (26.8)	66 (53.7)	13 (10.6)	5 (4.1)	6 (4.9)
Age						
24–39	101 (38.1)	30 (29.7)	54 (53.5)	10 (9.9)	3 (3.0)	4 (4.0)
40–54	89 (33.6)	34 (38.2)	44 (49.4)	7 (7.9)	2 (2.2)	2 (2.2)
55–67	75 (28.3)	23 (30.7)	31 (41.3)	11 (14.7)	2 (2.7)	8 (10.7)
Educational level						
Nonspecialist	73 (27.5)	20 (27.4)	40 (54.8)	11 (15.1)	2 (2.7)	0 (0.0)
Specialist^a^	192 (72.5)	67 (34.9)	89 (46.4)	17 (8.9)	5 (2.6)	14 (7.3)

a The specialist training for neurologists in Sweden is 5 years, following the 5.5 years of basic training and the 2 years of internship.

*Frequencies of different situations*: “How often in your clinical work do you…?” (23 items) with the same response alternatives as above, and “How often do you experience lack of time…?” (3 items) with response alternatives: Every day/About once a week/About once a month/A few times a year/Never or almost never. The response alternatives were categorized into three groups (Tables 2 and 3).

**Table 2:** Proportion of neurologists (*n* = 251) reporting frequency of different situations regarding sickness certification

	Items	At least once a week	About once a month or a few times per year	Never or almost never
	**When handling sickness certification tasks, how often do you ** ***not*** **have enough time…**			
	With your patients?	67.1	26.1	6.8
	To manage patient‐related aspects (e.g., issuing certificates, contacting other stakeholders, documentation, and meetings)?	78.7	17.3	4.0
	For further education, supervision, or reflection?	63.1	26.6	10.2
	**How often in your clinical work do you…**			
	Find it problematic to handle sickness certification?	35.9	57.6	6.5
	Experience that your competence in insurance medicine is not sufficient?	10.1	69.1	20.7
	Experience sickness certification consultations to be a work environment problem?	21.8	48.6	29.6
	Have time scheduled, alone or with colleagues, for supervision, feedback, or reflection regarding sickness certification issues?	0.4	16.9	82.7
	Write other types of certificates e.g., for applications regarding disability pension?	18.0	72.4	9.6
**Patient‐related aspects**	Encounter a patient who wants to be on sick leave for some other reason than work incapacity due to disease or injury?	7.7	67.0	25.4
	Have patients saying no, partly or completely, to a sick leave you suggest?	1.2	58.4	40.3
	Say no to a patient who wants a sickness certificate?	4.0	81.9	14.1
	Experience conflicts with patients about sickness certification?	3.6	62.1	34.3
	Worry that a patient will report you regarding sickness certification?	0.0	10.0	90.0
	Feel threatened by a patient in connection with sickness certification?	0.0	9.3	90.7
	Worry that patients will go to another physician if you don't issue a sickness certificate?	0.0	5.2	94.8
	Have patients saying that they will change physician if you don't issue a sickness certificate?	0.0	7.2	92.8
	Issue sickness certificates to patients without seeing them (e.g., by telephone)?	23.2	64.0	12.8
**Collaboration‐related aspects**	Or your health care team participate in coordination meeting with social insurance and/or employer regarding sickness certified patients?	2.8	47.6	49.6
	Or your care team have contact with employers in ways other than via the coordination meetings?	0.0	33.1	66.9
	Refer patients to occupational health services?	0.0	55.6	44.4
	Collaborate with or refer patients to a counsellor/psychologist in sickness‐certification cases?	10.4	63.9	25.7
	Collaborate with or refer patients to physical or occupational therapists in sickness‐certification cases?	15.6	66.8	17.6
	Confer with other physicians when handling cases involving sickness certification?	3.2	63.2	33.6
	Have contact with social services regarding sickness‐certification cases?	0.0	17.2	82.8
	Have contact with the employment offices regarding sickness‐certification cases?	0.4	53.6	46.0
	Lack someone (e.g., coach or case manager) who coordinates measures for the patients?	14.1	55.7	30.1

**Table 3:** Proportions of board‐certified specialist (*n* = 178) and nonspecialist (*n* = 73), respectively, working in neurological clinics, regarding different situations concerning sickness certification, and *p*‐values for differences between the two groups

	Items	Nonspecialist		Specialist		
		At least once a week	Never or almost never	At least once a week	Never or almost never	*p*‐value
	**When handling sickness certification tasks, how often do you experience lack of time…**					
	to manage patient‐related aspects?	74.0	6.8	80.7	2.8	.018
	for further education, supervision, or reflection?	57.1	14.3	65.5	8.6	.017
	**How often in your clinical work do you…**					
	experience that your competence in insurance medicine is not sufficient?	11.3	7.0	9.7	26.3	.004
	write other types of certificates e.g. for applications regarding disability pension?	8.2	19.2	22.0	5.6	.000
Patient‐related aspects	experience conflicts with patients about sickness certification?	4.2	23.6	3.4	38.6	.031
	feel threatened by a patient in connection with sickness certification?	0.0	84.9	0.0	93.1	.043
	issue sickness certificates to patients without seeing them (e.g., by telephone)?	15.1	20.5	26.6	9.6	.008
**Collaboration‐related aspects**	or your healthcare team participate in coordination meetings with social insurance and/or employer regarding sickness certified patients?	0.0	72.6	4.0	40.1	.000
	or your healthcare team have contact with employers in ways other than via the coordination meetings?	0.0	83.3	0.0	60.2	.001
	refer/send patients to occupational health services?	0.0	60.3	0.0	37.9	.002
	confer with other physicians when handling cases involving sickness certification?	6.8	13.7	1.7	41.8	.000
	have contact with the employment offices regarding sickness‐certification cases?	0.0	72.6	0.6	35.0	.000
	lack for someone (e.g. a coach or case manager) who coordinates measures for the patients?	21.1	22.5	11.4	33.1	.041
	**How problematic do you generally find it to…**	**Very**	**Fairly**	**Very**	**Fairly**	
	assess the optimum duration and degree of sickness absence?	18.1	47.2	8.6	41.4	.008
	make a plan of action or of measures to be taken during the sick leave?	16.4	34.2	7.5	29.3	.006
	make a long‐term prognosis about the future work capacity of patients on sick leave?	31.5	39.7	18.3	37.7	.010
	consider, together with the patient, possible lifestyle and life situation changes?	12.3	38.4	6.3	17.8	.000
	discuss and know how to deal with other psychosocial problems when handling a patient on sick leave?	13.9	41.7	3.5	30.1	.000
	handle situations in which you and a patient have different opinions about the need for sick leave?	19.7	45.1	4.1	25.1	.000
	handle situations in which you and other members of the healthcare team have different opinions about sickness certifying a patient?	0.0	9.7	2.3	4.1	.002
	handle long‐term sickness certifications (91–180 days)?	25.0	44.4	18.3	34.9	.002
	handle very long‐term sickness certifications (>180 days)?	41.1	24.7	23.1	28.3	.000

**Figure 1:**
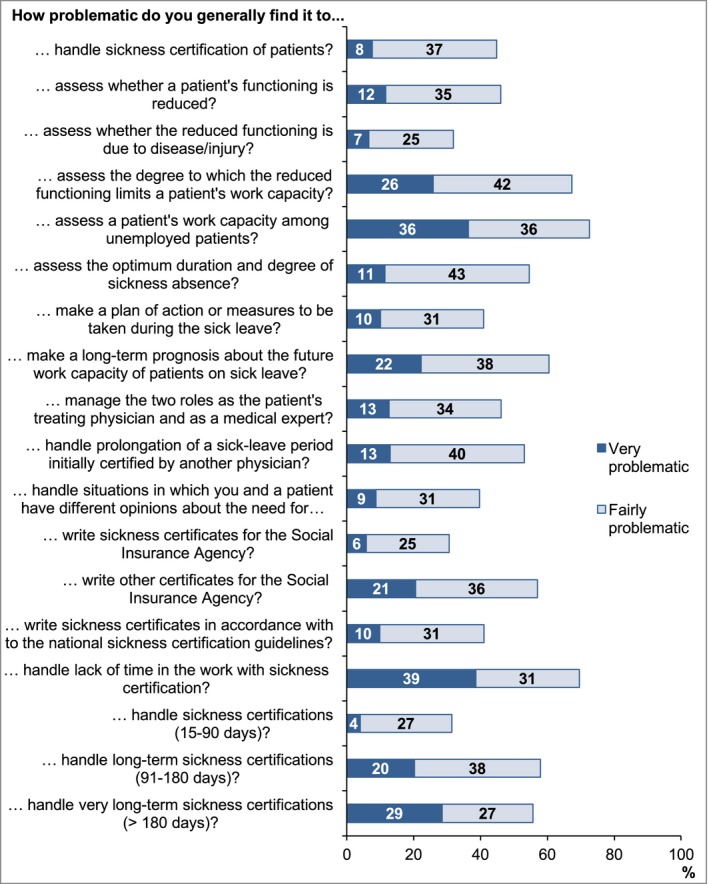
Proportion of neurologist (*n* = 251) rating different aspects of sickness certification as very or fairly problematic

*Severity of different problematic situations*: “How problematic do you generally find it to…?” (18 items) with response alternatives: Very/Fairly/Somewhat/Not at all (Figure 1).

**Figure 2:**
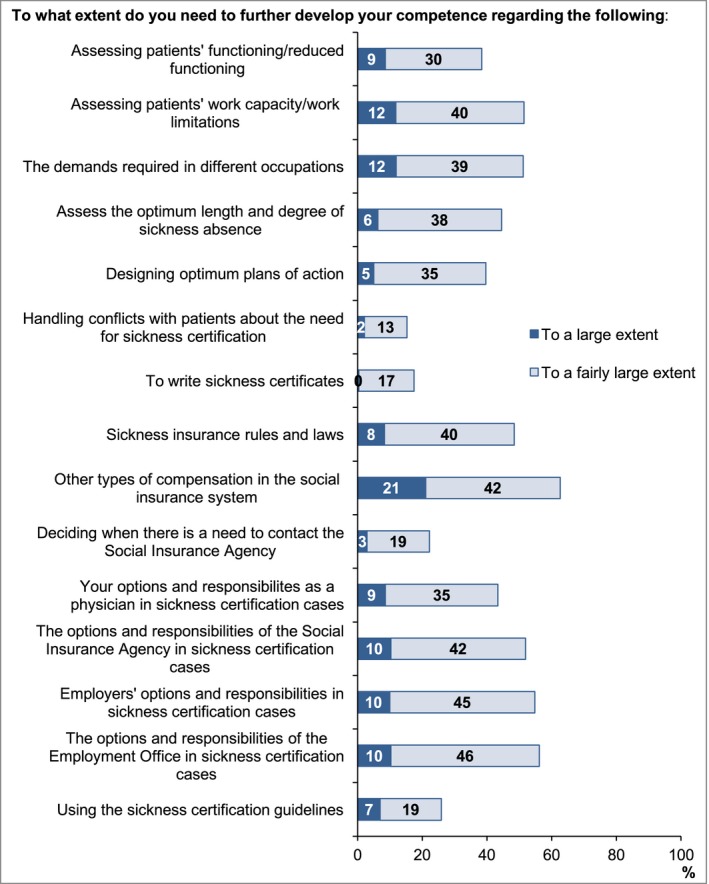
Proportion of neurologist (*n* = 251) reporting a large or very large need to further develop their competence concerning specific issues related to sickness certification

*Need for more competence*: “To what extent do you need to further develop your competence in relation to the following?” (15 items) with response alternatives: To a large extent/To a fairly large extent/To some extent/Not at all (Figure 2).

The mean nonresponse rate on specific questions was 0.9% for questions in Table 2, 1.5% in Table 3, 2.2% in Figure 1, and 3.1% in Figure 2.

The results are presented with descriptive statistics. Mann–Whitney U‐tests were used to compare specialist with nonspecialist.

The Regional Ethical Review Board in Stockholm approved the project.

## 3 RESULTS

A large majority (95%) of the 265 responding neurologists had sickness certification consultations at least a few times per year, and one third at least six times per week (Table 1). Among the 251 who had such consultations, a third found it problematic to handle sickness certification and 22% experienced sickness certification consultations as a work environment problem at least once a week (Table 2). Even though two‐thirds experienced conflicts with patients regarding sickness certification at least a few times per year, the vast majority did not feel threatened or worried about a patient taking action against them in connection to sickness certification (Table 2).

Eighty‐three percent never or almost never had time scheduled for supervision, feedback, or reflection regarding insurance medicine issues and 63% lacked time for further education, supervision, or reflection at least once a week (Table 2). Moreover, 70% found it very or fairly problematic to handle lack of time regarding sickness certification (Figure 1). Half of the neurologists never or almost never participated in coordination meetings with employers and insurance officers regarding sick‐listed patients and two thirds never or almost never had contact with employers other than via coordination meetings (Table 2). There were large differences between specialists and nonspecialists regarding the last two questions (Table 3).

The tasks that were experienced as very or fairly problematic by most neurologists were; assessing the degree to which the reduced functioning limits a patient's capacity to perform work assignments (67.3%); especially for unemployed patients (72.6%); and making long‐term prognosis regarding the future work capacity (60.5%) (Figure 1). A larger proportion among nonspecialists than among specialists, experienced these issues as very or fairly problematic (Table 3).

Overall, the neurologists expressed an interest to improve their competence regarding sickness certification. Eighty‐four percent stated a need for competence to a large or fairly large extent for at least one of the issues presented in Figure 2. Also, 79% frequently experienced that their competence in insurance medicine was insufficient, 10% as often as once a week (Table 2). Several (63%) expressed a need for more knowledge regarding also other types of compensations in the social insurance system (Figure 2). A higher rate of the nonspecialists than the specialists stated a need to further develop their competence in insurance medicine (Table 4).

**Table 4:** Proportions of board‐certified specialist (*n* = 178) and nonspecialist (*n* = 73), respectively, working in neurological clinics, regarding to what extent they needed further competence in insurance medicine, and *p*‐values for differences between the two groups

Items	Nonspecialist		Specialist		
To what extent do you need to further develop your competence in relation to the following?	To a large extent	To a fairly large extent	To a large extent	To a fairly large extent	*p*‐value
Assess patients' functioning/reduced functioning	12.7	40.8	6.9	25.3	.000
Assess patients' work capacity	18.3	49.3	9.2	35.6	.000
Assess the optimum length and degree of sickness absence	8.5	49.3	5.2	33.7	.004
Design optimum plans of action	10.1	42.0	2.9	31.6	.007
Your options and responsibilities as a physician in sickness certification cases	12.7	42.3	6.9	31.8	.012
Handling conflicts with patients about the need for sickness certification	2.9	17.1	1.7	11.6	.006
Other types of compensation in the social insurance system	29.6	47.9	17.4	39.0	.001
Decide when there is a need to contact the Social Insurance Agency	8.5	28.2	0.6	15.7	.001
Employers' options and responsibilities in sickness certification cases	11.3	54.9	9.3	40.7	.030

## 4 DISCUSSION

To the best of our knowledge, this is the first study that, at a detailed level, elucidates neurologists' experiences of sickness certification tasks. We found that sickness certification is a common task among neurologists and that they frequently experience a variety of problems associated with this task. Nevertheless, the prerequisites for neurologists to develop, maintain, and practice competence in insurance medicine were limited within their organizational setting. One of five even experienced this as a work environmental problem, as often as every week.

The neurologists rarely experienced conflicts with patients and a large majority never felt threatened or worried about patients taking actions against them in connection with sickness certification. Not to forget, for the 10% who actually experienced this, support is warranted. Even so, the results could be due to a shortage of neurologists in Sweden; why changing to another neurologist is not an option for most patients (Lokk, 2011). Moreover, as many of the neurological diseases are chronic in nature it may be desirable with a long‐term relationship between the patient and the physician to create a common understanding of the issues related to the individual's sickness certification.

About a third of the neurologists (35.9%)stated that they, on a weekly basis, found it problematic to handle sickness certification. This is a somewhat higher rate than among all sickness certifying physicians in Sweden that year (31.7) (Ljungquist et al., 2015).

A task that many neurologists experienced as problematic was assessing the degree to which reduced function limits a patient's work capacity (68%), especially for unemployed patients (73%) (Figure 1). The corresponding rates for all physicians were 58% and 64%, respectively (Ljungquist et al., 2015). For patients with a neurological diagnosis there is often a psychological impact to consider, and effects of such on work capacity is often more difficult to access which could be one reasons for why neurologists find it more difficult than other physicians to assess work capacity (Aghaei, Karbandi, Gorji, Golkhatmi, & Alizadeh, 2016; Balasooriya‐Smeekens, Bateman, Mant, & De Simoni, 2016). The results regarding problems with assessing patients' work capacity are in line with results from other studies (Bränström et al., 2014; Kedzia et al., 2015; Ljungquist et al., 2015; Lofgren et al., 2007). Physician's knowledge regarding specific work demands of patients is often very limited (Stigmar, Ekdahl, & Grahn, 2012) and even when good, most expressed lacking an instrument to assess work capacity. Also, most did not have contacts with patients' employers (Table 2). According to previous studies, physicians instead often rely on patients' self‐reports of working conditions rather than obtaining information direct from the employer (Edlund & Dahlgren, 2002; Pransky, Katz, Benjamin, & Himmelstein, 2002). Limited knowledge about work demands might delay the return to work since lack of workplace information gives less opportunity to discuss adjustments of work tasks in relation to the patient's current function. In patients seeking work, assessing the level of work incapacity becomes even more difficult, as the patient's work capacity then must be determined in relation to all available types of jobs.

Another problematic issue was to make a long‐term prognosis about the future work capacity for patients on sick leave. Prognoses for recovery are based on several factors, such as the severity of the injury/disease and the specific work tasks of the patient. If a disease also involves a risk for relapse it might be even more difficult to determine the progression of the disease and future impact on work capacity.

A majority of the neurologists experienced their competence in insurance medicine as insufficient, nonspecialists to a higher degree than specialists (Table 3). In all, 84% of the responding neurologists reported a need for more competence concerning sickness certification. At the same time, the majority responded that they did not have enough time to develop such competence, e.g., 83% stated never having scheduled time for supervision/feedback or reflection with colleagues regarding sickness certification issues.

Nonspecialists working in neurological clinics perceived sickness certification tasks as more problematic and reported a larger need for more competence regarding these tasks than the specialists. The result may be explained by the specialists' greater experience and knowledge of the patient allowing for perceived security in the assessments included in the task. The fact that the specialist to a higher degree perceived lack of time for the task may be related to that they handle the more complex cases.

To assess work capacity is a very complex task. Currently, in many countries there are attempts to develop instruments for this. Our results clearly show that the current focus on GPs in studies of physician's sickness certification need to be widened to also include neurologists. Managers of neurology clinics need to recognize the need of administrative prerequisites such as time and routines for training, collaboration, etcetera.

Strengths of this study are that all physicians working in neurology clinics in all of Sweden were invited, the large number of participants, and the many detailed questions regarding the studied issues. The high response rate, compared to most studies of physicians, is another strength. Nevertheless, the drop out is still a limitation and we do not know how those not participating would have answered the questions. As in all surveys, the participants can have interpreted the questions in different ways despite previous validations. Therefore, in this explorative study, the results should be interpreted with caution. Nevertheless, this is the so far, without comparison, largest study of neurologist's work with sickness certification.

## 5 CONCLUSIONS

Many neurologists experience sickness certification tasks as problematic and have limited resources for optimal work with such tasks, in terms of time, supervision, etcetera. The majority want to increase their competence in insurance medicine, specifically regarding assessment of work incapacity and social security aspects, and opportunities for this should be provided.

## CONFLICT OF INTEREST

The authors declare no financial or other conflicts of interest.
